# Tea Tasting

**Published:** 1872-01

**Authors:** P. H. Hayes


### TEA TASTING.

P. H. HAYES, M. D.

A New York correspondent of a Western paper has given the following particulars of the effects of "*Tea-tasting*" and sampling upon the constitutions of those engaged in it.

"The death of a famous tea broker in this city lately, calls to mind the curious nature of his business. I wonder if your readers know, that their fastidiousness in the choice of the herb which "cheers but not inebriates," is the cause of the establishment of a profession called *tea-tasting*, which is as certain death to a man as the habit of opium-eating. It is the business of the tea-tasters, to overhaul the cargoes of tea, classify and determine the value of each sort. In doing this, he first looks at the color of the leaf, and the general cleanliness of it. He next takes a quantity of the herb in his hand, and breathing his warm breath upon it, he snuffs up the fragrance. Then sitting down at the table in his office, on which is a long row of little porcelain cups and a pot of hot water, he 'draws' the tea, and tastes the infusion. In this way he classifies the different sorts to the minutest shade, marks the different prices, and is then ready to compare his work with the invoice. The skill of these tasters is truly marvelous, but the effect of the business on their health is, as I have said, ruinous: they grow lean, nervous and consumptive. At the end of a hard day's work they feel and act as fidgety and cross as a hysterical, old—bachelor!"

An English surgeon says: A physician called upon me in the middle of the day. It so happened that I answered the door myself, and as he entered, he appeared to me to be actuated by great terror, and upon my asking him what was the matter, he said, I have called to request you would let me in and allow me to die in your house. When he sat down I examined his pulse, which was irregular and so feeble as scarcely to be found. He said he had called at the house of Dr. H—— and then at the house of Dr. P——, but finding neither at home he had come to mine, where he entreated I would allow him to expire, which event he was sure was inevitable. I cannot tell at this distance of time, what circumstance it was which made me ask him if he had been drinking strong green tea, and he immediately replied that he had drank a great deal during the whole of the preceding night, which he had spent watching with an uncle.

A woman past middle life, of whom I knew, took her green tea six times a day. Her general health appeared to be good, but when the time came for her tea, if it was delayed, she soon became too weak to make it herself, she also became rapidly depressed in spirits, even irritable and passionate, but as soon as she could get her tea, she was "all right again."

Another woman, and the mother of a small family, came under my care with a miserable dyspepsia, and bowed over with pains and cramps in her stomach. She had been in the habit of drinking her green tea four times or more in the day, and she could not digest her food without it. She said "always when the strength of the tea is going off, I am cross, and I sometimes feel cross enough to my children to tear them to pieces. If work goes wrong, I get perfectly crazy over it."

One of the physicians of the Bellevue Hospital in New York, told me that a great many servant girls enter the hospital with a wretched form of dyspepsia, hard to treat and caused by using freely strong tea and coffee, and taking very little food. The first thing they complain of is a pain in the heart, and palpitation and their chief fear is heart disease.

Rev. Mr. B——, a spare and nervous man came into my office for professional advice. Putting my ear to his chest I found his heart enlarged and dilated, and it was painful to listen to its rapid and heavy beating.—He was a musician and very fond of his violin. He admitted that he was "tied up" to his tea and coffee, and he strongly protested that he could not leave them off, and he said I must not insist upon it, for if I did he could not play his *Sonatas*.

In another instance I failed to convince a clergyman that his sallow paleness, his unsteady hand, his tremors, sighings, wakefulness and palpitation, his weak digestion and fear of insanity, were owing to his driving his brain on strong tea, drank three times a day. With such a habit as this no man can ever be in a truly natural state of mind or body. While the strength of the stimulant lasts, his vivacity, his quick flowing ideas,his lively emotions,his rapid movements, his feelings of exhilaration and exultation, are as unnatural, as the lassitude, fretfulness and gloom which follow. These persons cannot account for their own moods or "ups and downs" in mind and body. I have heard one of them say, it was marvelous to him, how differently the same work, or enterprise would look to him at different times in the same day.

Few persons can be found who do not know that tea and coffee exhilarate,even flush the face, and sometimes excite the whole frame, but they never seem to have thought that by an inevitable law of compensation, there must come some degree of recoil, some counterpart of depression to such excitement.

Multitudes of American women seem to become dependent upon tea and coffee, to abate the languor and lassitude consequent upon passing so large a part of their whole lives within doors, and for one-half the year in this climate in rooms heated by air-tight stoves. In this way, nervousness is grafted upon bodily debility, and a type of invalidism established, now so common and so well understood by physicians, as completely to have revolutionized the practice of forty years ago. Literary and sedentary men are perhaps even more dependent upon these, if not stronger stimulants, to compensate in a bad way for the want of more sunlight, fresh air, exercise and physical recreation. If there be a temperate use of tea and coffee, it may nevertheless be safely asserted that they are always injurious to children, and young persons, and to all others of weak digestion. Yet persons of weak digestion are the very ones to crave these stimulants, and finally to suffer in consequence from morning headache, trembling of the limbs, sudden "giving out" faint spells, cold hands and feet, all of which and many other strange feelings are apt to come over those who have to wind up on tea or coffee, three times a day, to keep their brain and nerves in business or social aptitude.

In all such cases, the power of the body to keep itself warm by its own internal vital action, is diminished. These persons not only have actually far less animal, or bodily warmth, but they are besides nervously sensitive to cold, and for both these reasons, take cold easily, and the great prevalence of colds, cough, catarrh, rheumatism and neuralgia must be set down in great part to the causes I have asserted.

